# Genome Sequence of *Kocuria palustris* Strain W4

**DOI:** 10.1128/genomeA.00074-16

**Published:** 2016-03-31

**Authors:** Jakob Herschend, Prem K. Raghupathi, Henriette L. Røder, Søren J. Sørensen, Mette Burmølle

**Affiliations:** Section for Microbiology, Department of Biology, University of Copenhagen, Copenhagen, Denmark

## Abstract

We report the 3.09 Mb draft genome sequence of *Kocuria palustris* W4, isolated from a slaughterhouse in Denmark.

## GENOME ANNOUNCEMENT

*Kocuria palustris* is a spherical, aerobic Gram-positive, nonmotile bacterium belonging to the family *Micorcoccaceae*, phylum *Actinobacteria* (1). Though the bacteria of this family are very commonly isolated from various ecological niches, the amount of information on this species is inadequate. Currently, there is only one publically available draft genome assembly of *K. palustris* PEL (2). Here, we present the draft assembly of *K. palustris* W4, isolated from a slaughterhouse in Denmark (3).

The sequence libraries of *K. palustris* W4 were prepared using the Nextera XT kit (Illumina, USA), according to the manufacturer’s recommendations, followed by sequencing as a part of the flowcell, as 2 × 250-base paired-end reads, using Illumina MiSeq (Illumina, USA) technology. The reads were cleaned and trimmed using CLC Genomics Workbench 7 (CLC bio, Denmark) and assembled in SPAdes 3.5.0 (4). The assembled genome was annotated using the RAST server (5) and RNAmmer 1.2 (6) to screen for noncoding rRNAs and tRNAs. The draft genome of *K. palustris* W4 contains 95 contigs with an average G+C content of 69.9% at a coverage of 179× and 51 predicted RNA genes. RNAmmer analysis predicted 3 copies of 5s and 1 copy each of 23s and 16s rRNA genes.

A total of 2,732 coding regions were found, out of which 1,225 were functionally annotated. Compared to the already available genome of the species *K. palustris* PEL that has 42 genes involved in virulence, diseases, and defense and a further 25 genes for resistance against antibiotics and toxic compounds, the annotated genome of W4 has 49 genes involved in virulence, diseases, and defense, including 33 genes for resistance against antibiotics and other toxic compounds. Further, strain W4 has 88 stress response genes, whereas strain PEL has only 69 genes in this category. This difference may reflect the adaptation of strain W4 to survive in hostile industrial environments. Functional comparison available on the RAST server revealed *Kocuria rhizophila* DC2201 to be the closest neighbor of *K. palustris* W4. A comparative genomic analysis between *Kocuria* strains will facilitate a better characterization of these strains.

### Nucleotide sequence accession numbers.

The whole-genome shotgun project for *Kocuria palustris* W4 has been deposited in the European Nucleotide Archive (ENA) under the accession numbers CZJR01000001 to CZJR01000095. The version described in this paper is the first version.

## References

[1] Kovács G, Burghardt J, Pradella S, Schumann P, Stackebrandt E, Màrialigeti K 1999 *Kocuria palustris* sp. nov. and *Kocuria rhizophila* sp. nov., isolated from the rhizoplane of the narrow-leaved cattail (*Typha angustifolia*). Int J Syst Bacteriol 49:167–173. doi:10.1099/00207713-49-1-167. 10028258

[2] Sharma G, Khatri I, Subramanian S 2014 Draft genome sequence of *Kocuria palustris* PEL. Genome Announc 2(1):e01261-13. doi:10.1128/genomeA.01261-13. 24504000 PMC3916494

[3] Røder HL, Raghupathi PK, Herschend J, Brejnrod A, Knøchel S, Sørensen SJ, Burmølle M 2015 Interspecies interactions result in enhanced biofilm formation by co-cultures of bacteria isolated from a food processing environment. Food Microbiol 51:18–24. doi:10.1016/j.fm.2015.04.008. 26187823

[4] Nurk S, Bankevich A, Antipov D, Gurevich AA, Korobeynikov A, Lapidus A, Prjibelski AD, Pyshkin A, Sirotkin A, Sirotkin Y, Stepanauskas R, Clingenpeel SR, Woyke T, McLean JS, Lasken R, Tesler G, Alekseyev MA, Pevzner PA 2013 Assembling single-cell genomes and mini-metagenomes from chimeric MDA products. J Comput Biol 20:714–737.doi:10.1089/cmb.2013.0084 24093227 PMC3791033

[5] Overbeek R, Olson R, Pusch GD, Olsen GJ, Davis JJ, Disz T, Edwards RA, Gerdes S, Parrello B, Shukla M, Vonstein V, Wattam AR, Xia F, Stevens R 2014 The SEED and the Rapid Annotation of microbial genomes using Subsystems Technology (RAST). Nucleic Acids Res 42:D206–D214. doi:10.1093/nar/gkt1226. 24293654 PMC3965101

[6] Lagesen K, Hallin P, Rødland EA, Staerfeldt H-H, Rognes T, Ussery DW 2007 RNAmmer: consistent and rapid annotation of ribosomal RNA genes. Nucleic Acids Res 35:3100–3108. doi:10.1093/nar/gkm160. 17452365 PMC1888812

